# Swarm Hunting and Cluster Ejections in Chemically Communicating Active Mixtures

**DOI:** 10.1038/s41598-020-62324-0

**Published:** 2020-03-27

**Authors:** Jens Grauer, Hartmut Löwen, Avraham Be’er, Benno Liebchen

**Affiliations:** 1 Institute for Theoretical Physics II: Soft Matter, Heinrich-Heine University Düsseldorf, Universitätsstraße 1, 40225 Düsseldorf, Germany; 2 Zuckerberg Institute for Water Research, The Jacob Blaustein Institutes for Desert Research, Ben-Gurion University of the Negev, Sede Boqer Campus, 84990 Midreshet Ben-Gurion, Israel; 3 Department of Physics, Ben-Gurion University of the Negev, Beer Sheva, 84105 Israel; 4 Institut für Festkörperphysik, Technische Universität Darmstadt, 64289 Darmstadt, Germany

**Keywords:** Biological physics, Condensed-matter physics, Statistical physics

## Abstract

A large variety of microorganisms produce molecules to communicate via complex signaling mechanisms such as quorum sensing and chemotaxis. The biological diversity is enormous, but synthetic inanimate colloidal microswimmers mimic microbiological communication (synthetic chemotaxis) and may be used to explore collective behaviour beyond the one-species limit in simpler setups. In this work we combine particle based and continuum simulations as well as linear stability analyses, and study a physical minimal model of two chemotactic species. We observed a rich phase diagram comprising a “hunting swarm phase”, where both species self-segregate and form swarms, pursuing, or hunting each other, and a “core-shell-cluster phase”, where one species forms a dense cluster, which is surrounded by a (fluctuating) corona of particles from the other species. Once formed, these clusters can dynamically eject their core such that the clusters almost turn inside out. These results exemplify a physical route to collective behaviours in microorganisms and active colloids, which are so-far known to occur only for comparatively large and complex animals like insects or crustaceans.

## Introduction

Chemotaxis - the movement of organisms in response to a chemical stimulus - allows them to navigate in complex environments, find food and avoid repellants. It is involved in many biological processes where microorganisms (or cells) coordinate their motion; these include wound healing, fertilization, pathogenic invasion of a host, and bacterial colonization^1,2^. In such cases, microorganisms are attracted (or repelled) by certain substances (chemoattractants/ chemorepellents), but they are also attracted to chemicals produced by other microorganisms (or cells), such as cAMP in the case of Dictyostelium cells^3^ or autoinducers in signaling Escherichia coli^4^, which leads to chemical interactions (communication) among the microorganisms.

**Figure 1:**
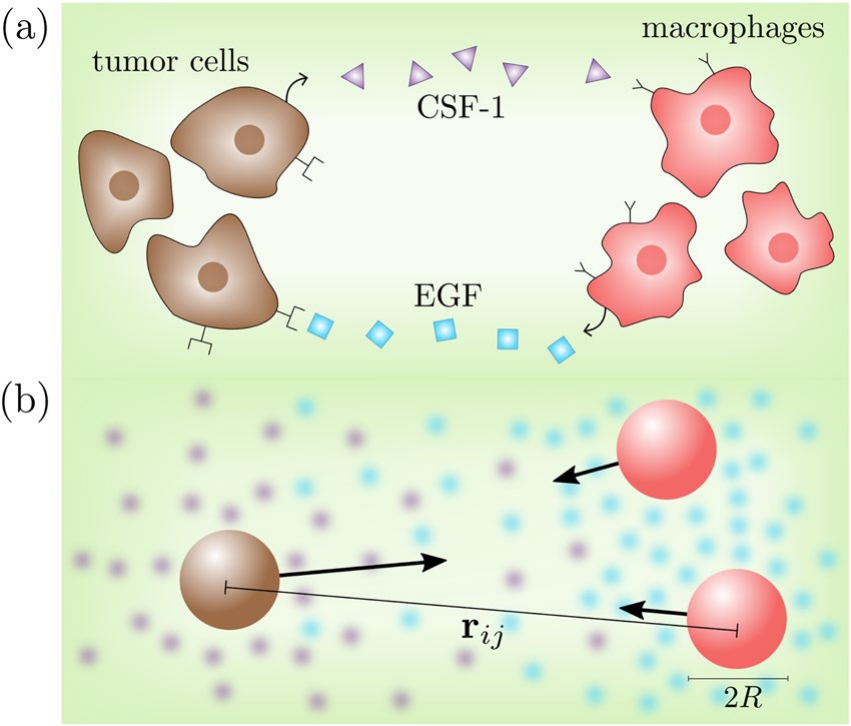
Schematic: (**a**) Interaction between tumor cells and macrophages (**b**) physical minimal model used in the simulation: two species realized as different particles (brown and red) with radius $R$ and distance ${{\bf{r}}}_{ij}$. The movement of the particles depends both on their self-produced chemicals (blue and purple) and on the concentration produced by the other species. Arrows represent effective chemical interactions among the particles, which in general are non-reciprocal.

While many existing models studying microbiological chemotaxis (or chemical interactions) focus on a single species^5–12^, the typical situation in the microbiological habitat is that various different species simultaneously produce certain chemicals to which others respond via chemotaxis or based on quorum sensing mechanisms. One simple example involving chemical signaling across species is provided by macrophage-facilitated breast cancer cell invasion which has recently been modeled^13^. There, tumor cells attract macrophages, which are certain white blood cells normally playing a key role in the human immune system. They then control the physiological function of the macrophages and exploit their abilities. More specifically, the tumor cells produce the colony-stimulating factor (CSF-1) leading to the attraction and growth of macrophages which in turn release epidermal growth factors (EGF) resulting in the growth and mobility increase of the tumor cells (see Fig. 1).

Similarly to microorganisms, synthetic inanimate colloids, coated with a material which catalyzes a certain reaction on (a part of) their surface, show chemical interactions as well^14–16^. There, the colloids act as sources of the chemical field, which shows a 1/$r$-steady-state far-field profile in 3D (if the chemical does not ’decay’ e.g. through bulk reactions), leading to long-ranged chemical interactions between the colloids. For active colloids^17–21^, these interactions have been explored in single-species systems^22–27^, and more recently also in mixtures^28–34^, where chemical interactions can be non-reciprocal and break action-reaction symmetry^28,35,36^. This allows for the formation of active molecules^28–30^, where self-propulsion spontaneously emerges when the underlying nonmotile ’colloidal atoms’ bind together. Similarly as for their microbiological counterparts, in all these studies on mixtures of synthetic colloids it has been assumed that the different species interact via a single chemical substance.

**Figure 2:**
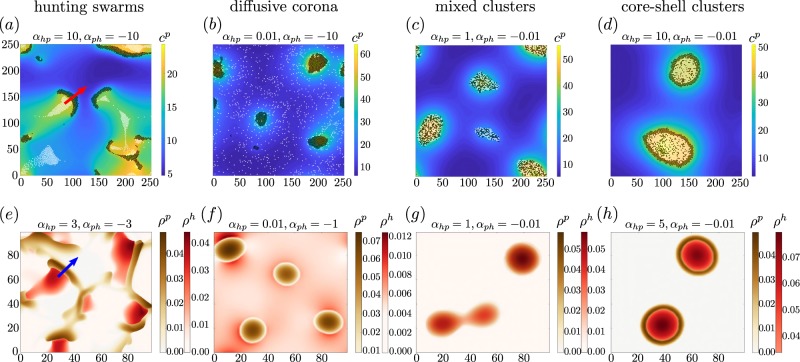
*Hunting swarms* and *core-shell clusters*: Simulation snapshots of Eqs. (3), (4) for $2N=2000$ chemically interacting particles (white dots represent hunter-particles; black dots show prey-particles) coupled to self-produced chemical fields at time $t=1500$ (a), 5000 (b-d). Panels (**a–d**) show particle based simulations, where colours show the chemical field produced by the prey ${c}^{p}$, panels (**e–h**) show simulations of the associated continuum equations at time $t=5000$ (e), 10000 (f-h), where colours show the density of hunters ${\rho }^{h}$ and prey ${\rho }^{p}$. (**a,e**) show *hunting swarm* patterns, (**c,g**) show *mixed clusters*, (**b,f**) show *core-shell clusters* with diffusive and (**d,h**) with rigid corona. Dimensionless parameters (tildes omitted): ${\alpha }_{pp}=1$, ${\alpha }_{hh}=0$, $\mu =0.001$, ${D}_{c}=1$, $D=0.001$ (a-d), $D=0.01$ (e-h) for reasons of stability, $\epsilon =1$ and box length ${L}_{{\rm{box}}}=250$ (a–d), ${L}_{{\rm{box}}}=100$ (e-h). See supplementary material for simulation details and the stabilization method used for the field equations underlying panels (e–h).

In the present work, we propose and explore a physical minimal model for two species of chemically interacting particles, both of which produce an individual chemical substance. Such a situation occurs for example in tumor-macrophage systems involving the EGF/CSF-1 paracrine signalling loop between two cell types mentioned above^13^. By comparing numerical simulations of Langevin equations describing the particle dynamics (Fig. 2(a–d)) with numerical solutions of deterministic continuum equations describing the dynamics of their density fields (Fig. 2(e–h)) and a linear stability analysis, we systematically explore and analyze the phase diagram of this system. As our key result, we discover a “hunting-swarm phase” (see Fig. 2(a,e)), where both species segregate and form individual swarms, one of them closely pursuing the other one. This phase resembles a group of hunters chasing a group of prey trying to stay together, not allowing the hunters to split up the group. It is interesting to note that a phenomenologically similar form of swarm hunting also occurs in much larger systems, e.g. in insects and systems of larvae hunting crustaceans (Daphnia)^37–39^, where collective predation phenomena and escape strategies have already been analyzed^40^, but not for microorganisms or synthetic colloids. Physically this phase occurs, if one species (“the hunters”) is attracted by the chemicals produced by the other species (“the prey”) and the prey is in turn repelled by the chemicals produced by the hunters. Note that a different form of moving clusters has recently been observed also in simulations involving only one chemical species^33^. Unlike the hunting swarms which we present here, the moving clusters in^33^ do not involve a species segregation into two individual swarms, but rather consist of a single aggregate of asymmetrically distributed predator and prey particles. By systematically exploring the parameter space underlying our model, we find that hunting swarms in fact occur generically if the chemical interactions are strong enough and have opposite sign. However, if the response of hunters and prey to the chemicals produced by the respective other species is strongly asymmetric, we instead find dense clusters of one species surrounded by a diffusive or rigid corona of particles from the other species (see Fig. 2(b,d,f,h)). These core-shell clusters can show a complex dynamics, ejecting their interior once they have formed. This behaviour hinges on model-ingredients which have not been considered in previous models of chemically interacting particle mixtures^31,33,41^. These are (i) a finite relaxation time of the chemicals leading to delay or memory effects in the absence of which the cluster ejections do not occur and (ii) the presence of two chemicals, which can lead e.g. to a coexistence of instantaneous and non-instantaneous interactions and in general also to coexisting attractions and repulsions with different ranges. The setup considered in the present work allows us to exemplify that a phenomenologically similar ejection may in principle originate from a remarkably simple mechanism hinging on a systematic invasion of the hunters into a cluster of prey particles, as we will later discuss in detail.

## Model

We consider an ensemble of two species of overdamped colloids (synthetic or biological), which we call prey and hunters, $s\in \{p,h\}$, each of which contains $N$ particles which produce a chemical field ${c}^{s}({\bf{r}},t)$ with a rate ${k}_{0}$. We assume that each particle responds to the chemical fields either via synthetic chemotaxis, which leads to a coupling $\propto \nabla {c}^{s}$ in far-field^22,27^, similar as for apolar colloids, or via biological chemotaxis which is sometimes modeled using an analogous form of the coupling^6^. To model the particle dynamics we use Langevin equations ($i=1,\ldots ,N$, $s\in \{p,h\}$):

1 $$\gamma {\partial }_{t}{{\bf{r}}}_{i}^{s}(t)=\sum _{s{\prime} \in \{p,h\}}{\alpha }_{ss{\prime} }\nabla {c}^{s{\prime} }-{\nabla }_{{{\bf{r}}}_{i}}V+\sqrt{2D}\gamma {{\boldsymbol{\eta }}}_{i}^{s}$$

where $D$ is the translational diffusion coefficient of the particles, $\gamma$ is the Stokes drag coefficient (assumed to be the same for both species) and ${{\boldsymbol{\eta }}}_{i}^{s}(t)$ represents unit-variance Gaussian white noise with zero mean. The chemotactic coupling coefficient of species $s$ to the chemical of species $s{\prime}$ is denoted as ${\alpha }_{ss{\prime} }$ where ${\alpha }_{ss{\prime} } > 0$ leads to chemoattraction and ${\alpha }_{ss{\prime} } < 0$ results in chemorepulsion (negative chemotaxis). In addition, $V$ accounts for excluded volume interactions among the particles which all have the same radius $R$ and which we model using the Weeks-Chandler-Anderson potential $V=\frac{1}{2}{\sum }_{i,j\ne i}{V}_{ij}$ where the sums run over all particles and where ${V}_{ij}=4\epsilon \left[{\left(\frac{\sigma }{{r}_{ij}}\right)}^{12}-{\left(\frac{\sigma }{{r}_{ij}}\right)}^{6}\right]+\epsilon$ if ${r}_{ij}\le {2}^{1/6}\sigma$ and zero else. Here $\epsilon$ determines the strength of the potential, ${r}_{ij}$ denotes the distance between particles $i$ and $j$, ${r}_{c}={2}^{1/6}\sigma$ indicates a cutoff radius beyond which the potential energy is zero and $\sigma =2R$ is the particle diameter.

The chemical fields ${c}^{h}({\bf{r}},t),{c}^{p}({\bf{r}},t)$ are produced by particles of hunters and prey, respectively. The dynamics of these fields, follows a diffusion equation (diffusion coefficient ${D}_{c}$), with additional (point) sources. We also use a sink term whose coefficient may be zero or nonzero if chemical reactions or other processes degrading the chemical occur in bulk. For simplicity we focus on the case where ${D}_{c},{k}_{0},{k}_{d}$ are identical for both species.

2 $$\begin{array}{ccc}{{\rm{\partial }}}_{t}{c}^{s}({\bf{r}},t) & = & {D}_{c}\Delta {c}^{s}-{k}_{d}{c}^{s}+{k}_{0}\mathop{\sum }\limits_{i=1}^{N}\delta ({\bf{r}}-{{\bf{r}}}_{i}^{s})\end{array}$$

To reduce the parameter space, we choose ${x}_{0}=R$ and ${t}_{0}=\frac{1}{{k}_{0}}$ as the units of length and time. The resulting dimensionless parameters are ${\widetilde{\alpha }}_{kl}=\frac{{\alpha }_{kl}}{\gamma {k}_{0}{R}^{d+2}}$, $\widetilde{\epsilon }=\frac{\epsilon }{\gamma {k}_{0}{R}^{2}}$,  $\widetilde{{D}_{c}}=\frac{{D}_{c}}{{k}_{0}{R}^{2}}$, $\widetilde{D}=\frac{D}{{k}_{0}{R}^{2}}$ and $\mu =\frac{{k}_{d}}{{k}_{0}}$ (see the Supplementary Material (SI) for details) and Eqs. (1), (2) reduce to (omitting tildes)

3 $$\begin{array}{ccc}{{\rm{\partial }}}_{t}{{\bf{r}}}_{i}^{s}(t) & = & \sum _{s{\rm{{\prime} }}\in \{p,h\}}{\alpha }_{ss{\rm{{\prime} }}}{\rm{\nabla }}{c}^{s{\rm{{\prime} }}}-{{\rm{\nabla }}}_{{{\bf{r}}}_{i}}V+\sqrt{2D}{{\boldsymbol{\eta }}}_{i}^{s}\end{array}$$

4 $$\begin{array}{lll}{\partial }_{t}{c}^{s}({\bf{r}},t) & = & \left({D}_{c}\Delta -\mu \right){c}^{s}+\mathop{\sum }\limits_{i=1}^{N}\delta \left({\bf{r}}-{{\bf{r}}}_{i}^{s}\right)\end{array}$$

## Hunting Swarms and Core-Shell Clusters

To explore the collective behaviour of many chemotactic agents, we now solve Eqs. (3) and (4) using Brownian dynamics simulations for the particle dynamics coupled to a finite difference scheme to calculate the dynamics of the self-produced chemical fields. We solve the diffusion equation in 2D for numerical efficiency and do not expect that our results would change qualitatively when solving the 3D diffusion equation (see the exemplaric simulation snapshot Fig. 2  in the SI and notice that the linear stability analysis which does not depend on the dimensionality of the diffusion equation is also in very good agreement with the particle based simulations). We use a quadratic simulation box with periodic boundary conditions (see SI for details) and observe the following patterns or nonequilibrium phases:

(i) A hunting swarm phase (see Fig. 2(a,e) and movies 1, 5), where both species segregate and form moving swarms which hunt each other.
(ii) A clustering phase (see Fig. 2(c,g) and movies 3, 7), where both species form a cluster and the different species are mixed.
(iii,iv) Two phases showing core-shell clustering, where one species forms the inner core and the other one forms a corona which may be diffusive (b,f) or rigid and which is strongly localized around the core (d,h).

Let us now characterize these phases and the dynamics leading to their emergence in detail.

**Figure 3:**
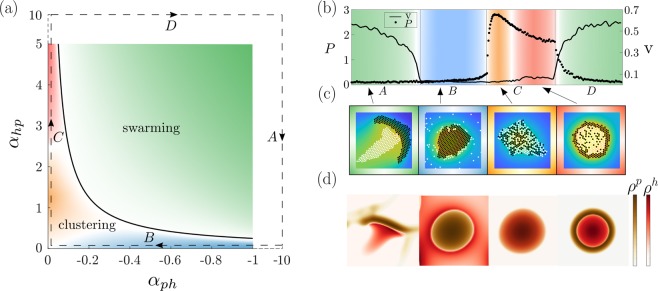
(**a**) State diagram in the plane spanned by the chemotactic cross coupling coefficients ${\alpha }_{ph}$ and ${\alpha }_{hp}$ for fixed ${\alpha }_{pp}=1$; ${\alpha }_{hh}=0$. The green domain represents hunting swarms, which are characterized by their ballistic motion and their emergence from an oscillatory instability (the black line shows the analytical prediction of the transition line), whereas colours for the remaining cluster phases are defined via the value of the mixing parameter shown in panel (b) (see text). The state diagram was created with more than 200 evaluated state points. (**b**) Mixing parameter $P$, counting the average number of black next neighbors per white particle and mean particle velocity $v$ at late times discriminating between the individual states: Each point corresponds to a parameter set on the dashed line in the parameter plane of panel a. The labels $A,B,C,D$ correspond to those shown on the dashed line in panel (a). (**c,d**) Extracts from the simulations underlying Fig. 2 (see movies 1–8).

To see in which parameter regimes each of these patterns prevails, in Fig. 3 we show a slice through the state diagram in the plane of the chemotactic cross-species coupling coefficients ${\alpha }_{ph} < 0$ and ${\alpha }_{hp} > 0$. Here we fix ${\alpha }_{pp}=1$ and ${\alpha }_{hh}=0$ so that prey-particles chemo-attract each other whereas the hunter-particles do not, but note that the specific values choosen here do not have much impact on the emerging patterns.

### Hunting swarms

The green area in Fig. 3(a,b,c) (movies 1, 5) represents the hunting-swarm phase which generically occurs if $| {\alpha }_{hp}{\alpha }_{ph}|$ is large enough, as we will later show using a linear stability analysis. Here the chemicals produced by the black-coloured particles in Fig. 2(a) (“prey”) attract the white coloured particles (“hunters”), whereas the hunter-produced chemicals repel the prey. This results in a swarm of “prey” pursued by a swarm of “hunters”. When two or more prey-swarms collide, the pursuing hunters produce a “cage” of high chemical density repelling the prey and trapping it temporarily in a small spatial domain. The prey then ’evades’ sidewards to escape from the hunter-fronts, forming new swarms moving perpendicular to the original ones (see movies 1, 5).

### Core-shell clusters

When decreasing ${\alpha }_{hp}$ (blue domain in Fig. 3(a,b) and movies 2, 6), so that the prey chemo-attracts the hunters only weakly, we observe that the prey aggregates and forms dense clusters, surrounded by a diffusive corona of hunters. Surprisingly, when staying with a large ${\alpha }_{hp}$ but decreasing ${\alpha }_{ph}$ instead (red domain in Fig. 3(a–c)), so that the hunters are strongly chemo-attracted by the prey, but the prey has only a weak tendency to avoid the hunter-produced chemicals, we see the opposite case: Although not attracting each other, the hunter-particles form a dense core, surrounded by the prey-particles (red domain in Fig. 3(c) and right panel of Fig. 3(d) and movies 4, 8). To see how these remarkable clusters emerge, let us explore the dynamics underlying their formation. Initially, the prey-particles, which chemo-attract each other aggregate and form very small clusters. While these clusters are forming, the aggregation of prey-particles locally increases the concentration of ${c}^{p}$ resulting in an attraction of hunter-particles, which directly invade the cluster, because $| {\alpha }_{hp}|  > | {\alpha }_{pp}|$. Consequently, as more and more hunters enter the cluster, the density of ${c}^{h}$ increases in the cluster center, repelling the prey. Since the prey-particles, in turn, couple stronger to their self-produced chemicals than to those produced by the hunters $| {\alpha }_{pp}|  > | {\alpha }_{ph}|$, they do not flee from the cluster but try to stay together. While in the simulations underlying Fig. 2, the hunters invade even small prey-clusters, for appropriate initial conditions, we can see a proper inside out reversal of comparatively large clusters (movie 9) (species reversal). In each case, the result is a counterintuitive pattern consisting of a dense cluster containing mostly hunters surrounded by ring of prey-particles.

### Dynamical ejections of particle clusters

**Figure 4:**
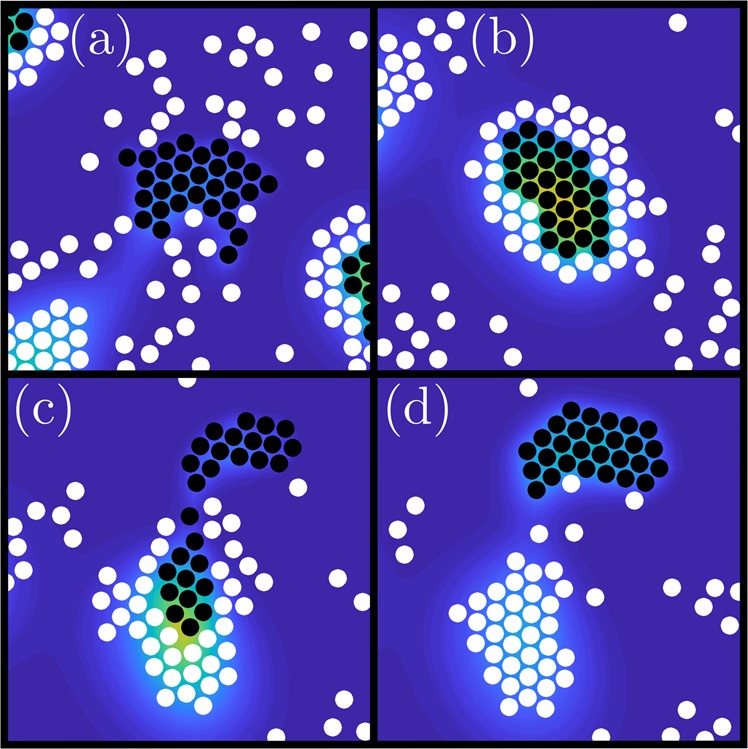
Sequence of simulation snapshots showing a cluster ejection which occurs due to chemical delay effects. Dimensionless parameters: ${\alpha }_{pp}=100$, ${\alpha }_{hh}=0$, ${\alpha }_{ph}=-1000$, ${\alpha }_{hp}=10$, ${\mu }^{p}=0.1$, ${\mu }^{h}=0.01$, ${D}_{c}^{p}=0.5$, ${D}_{c}^{h}=10$, $D=0.001$, $\epsilon =10$ with 500 prey-particles and 2000 hunter-particles.

We have investigated the dynamics of these core-shell clusters more precisely. Assuming the diffusion of ${c}^{p}$ is considerably lower than that of ${c}^{h}$ and ${c}^{h}$ is produced very slowly, this results in a certain delay effect. A typical course of this process is shown in Fig. 4 (see also movie 10). The prey-particles that attract each other initially accumulate and form clusters (Fig. 4(a)). Due to a resulting higher concentration of ${c}^{p}$, the hunters are also attracted. These hunter-particles then form a surrounding shell, but cannot immediately invade the prey-cluster as ${\alpha }_{pp} > {\alpha }_{hp}$ (Fig. 4(b)). Although slowly, the concentration of ${c}^{h}$ increases with time as more hunter-particles join. At some point a significant concentration of ${c}^{h}$ is exceeded and since $| {\alpha }_{ph}|  > | {\alpha }_{pp}|$, the prey-particles are ejected outwards from the center of the cluster (Fig. 4(c)). Since the chemicals ${c}^{p}$ produced by the prey diffuses on a much smaller timescale, the hunter-particles still move towards each other, form a dense cluster which persists for a while (Fig. 4(d)), before the hunter-cluster dissolves slowly and the whole process starts all over again.

### Irregular aggregation

Finally, when ${\alpha }_{ph}$, ${\alpha }_{hp}$ are both small, with $| {\alpha }_{ph}|  < | {\alpha }_{hp}|$ (orange regime in Fig. 3(a,b) and movies 3, 7), prey and hunter particles form clusters containing a seemingly irregular mixture of hunter and prey particles (Fig. 3(c), orange). These clusters emerge because we have a chemically mediated prey-prey attraction and a hunter-by-prey attraction which exceeds the prey-by-hunter-repulsion, so that effectively prey particles similarly strongly attract all other particles, leading to a rather irregular aggregation.

### Classification

In contrast to the static clusters, structures in the green region of Fig. 3(a) move ballistically and hence show a non-vanishing velocity. Figure 3(b) depicts the mean particle velocity $v(t)$ (see SI for details) at late times for parameters chosen along the dashed line in Fig. 3(a), where one can easily see how the velocity in regions of hunting swarms exceeds that in other regimes. While the hunting swarm phase, which emerges from an oscillatory instability, as discussed further below, can be clearly distinguished from the stationary cluster phases, let us define an “order parameter” $P$ to distinguish the remaining cluster phases. We define $P$ as the average number of black next neighbors (prey) per white particle (hunter), where we denote a neighbor as a particle within a distance ${r}_{ij} < 2+0.1$. Figure 3(b) shows $P$ for parameters chosen along the dashed line in Fig. 3(a). This parameter would have a value of 3 for completely irregular and infinitely large dense clusters. For the orange domain, where particles aggregate almost irregularly, it has a value $P > 2.5$, whereas red means ($1.5 < P < 2.3$) and blue means $P < 0.5$. Crossover regions between the individual patterns are marked by white domains in Fig. 3(a).

## Linear Stability Analysis – Emergence and Dynamics of Patterns at Early Times

To understand the structure of the state diagram we now introduce a continuum description for the particle dynamics and perform a linear stability analysis.

### Continuum model

The Smoluchowski equation, describing the dynamics of the (non-normalized) probability ${\rho }^{s}({\bf{r}},t)$ to find a particle of species $s$ at position ${\bf{r}}$ at time $t$ reads as follows ($s\in \{p,h\}$):

5 $${\partial }_{t}{\rho }^{s}=D\Delta {\rho }^{s}-\sum _{s{\prime} \in \{p,h\}}{\alpha }_{ss{\prime} }\nabla \cdot ({\rho }^{s}\nabla {c}^{s{\prime} }).$$

These deterministic equations are equivalent to the Langevin Equation (1) for point particles ($V=0$). We can now also rewrite the evolution equation for the chemical fields as follows:

6 $$\begin{array}{lll}{\partial }_{t}{c}^{s} & = & \left({D}_{c}\Delta -\mu \right){c}^{s}+{\rho }^{s}.\end{array}$$

Before carrying out a linear stability analysis, let us solve these equations numerically to test them: Integrating Eqs. (5), (6) for a uniform initial state (plus small fluctuations) on a square box of size ${L}_{box}=100$, we indeed find the same patterns as in our particle based simulations (Figs. 2(e–h) and 3) (see SI for details regarding these simulations and the used method to stabilize them).

### Linear stability analysis

We now linearize these four coupled equations around the stationary solution $(\rho ,c)=({\rho }_{0},{\rho }_{0}/\mu )$, which represents the uniform disordered phase, and solve them in Fourier Space, to understand the dynamics of a small plane wave perturbation with wavenumber $q$ around the uniform phase. We denote the dispersion relation of these fluctuations as $\lambda (q)$. If $\lambda$ has a positive real part for some $q$ value, the uniform phase is unstable. Calculating $\lambda$ (see Supplementary Material for details), we find that the uniform phase looses stability if

7 $$2D\mu  < {\rho }_{0}Re[{\alpha }_{pp}+\sqrt{4{\alpha }_{ph}{\alpha }_{hp}+{\alpha }_{pp}^{2}}],$$

where we have choosen ${\alpha }_{hh}=0$ as in our simulations. While Fig. 3 shows only parameter regimes where the uniform phase is unstable, we have performed additional simulations (see SI) which are in close quantitative agreement with the prediction of the onset of the instability due to Eq. (7). This holds true both in the regime where the instability is stationary and where it is oscillatory. The instability criterion shows that chemo-attractions among the prey particles support the emergence of a pattern in competition with diffusion and the potential decay of the chemical, whereas cross interactions only support the emergence of a pattern if they, ${\alpha }_{ph}$ and ${\alpha }_{hp}$, have the same sign.

To understand the transition between static clusters and hunting swarms, we also derive a criterion discriminating between stationary instability (static clusters, $\lambda$ is real) and oscillatory instabilities (moving structures, complex $\lambda$) which reads as follows (see SI):

8 $$2\sqrt{-{\alpha }_{ph}{\alpha }_{hp}} > {\alpha }_{pp}.$$

This criterion defines the solid black line in Fig. 3(a), which quantitatively agrees with our simulations. It shows that an oscillatory instability and hence moving patterns can appear only if ${\alpha }_{ph}$,${\alpha }_{hp}$ have opposite sign, i.e. if one species effectively hunts the other one, whereas the other one tries to escape. In this parameter regime where it is oscillatory, we have numerically tested the instability criterion (Eq. (7)) to see if it is shifted due to “perturbation convection”, see^42^. We did not find any shift, suggesting that the advective and absolute instability are very close to each other in the present case.

**Figure 5:**
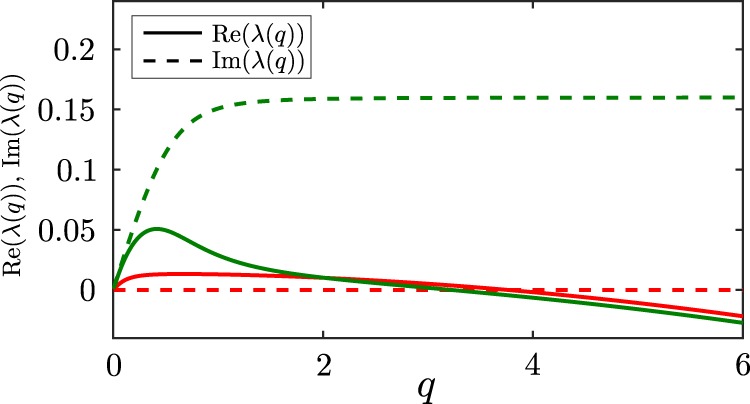
Real and imaginary part of the dispersion relation $\lambda$, for hunting swarms (green) and static clusters (red). Parameters as in Fig. 2(a,d).

In Fig. 5 we show the complete dispersion relation $\lambda (q)$ (real and imaginary part) of small plane wave fluctuations around the uniform phase. Here the location of the maxima in ${\rm{Re}}[\lambda (q)] > 0$ define the fastest growing mode, typically determining the length scale of the pattern at early times.

**Figure 6:**
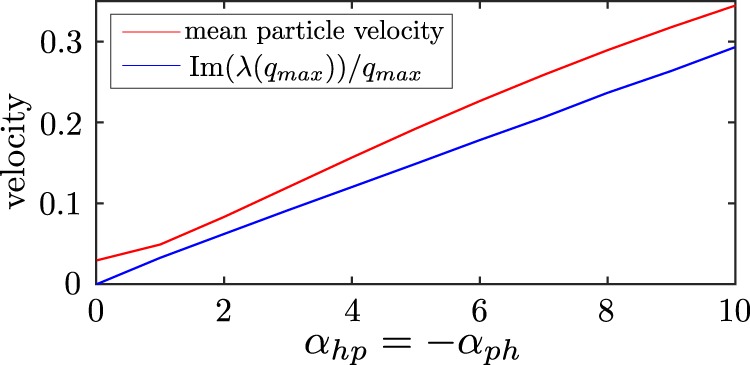
Mean particle velocity in the hunting swarm phase, extracted from the simulations underlying Fig. 3(a) at early times (red) and reduced imaginary part of $\lambda$ at the wavenumber corresponding to the fastest growing mode, i.e. ${\rm{Im}}\left(\lambda ({q}_{max})\right)$/${q}_{max}$ (blue) as a function of $-{a}_{ph}={a}_{hp}$.

Having understood the transition line between the cluster phases and the hunting swarms, let us also explore if we can understand how fast the swarms move. To do this, in Fig. 6, we compare the imaginary part of $\lambda$ (the expected speed of the hunting swarm is $v(q)=\frac{{\rm{Im}}(\lambda )}{q}\to \langle v\rangle \approx \frac{{\rm{Im}}\lambda ({q}_{max})}{{q}_{max}}$) with the velocity of the hunting swarms in our particle based simulations at early times and find close agreement.

## Structure and Growth at Late Times

**Figure 7:**
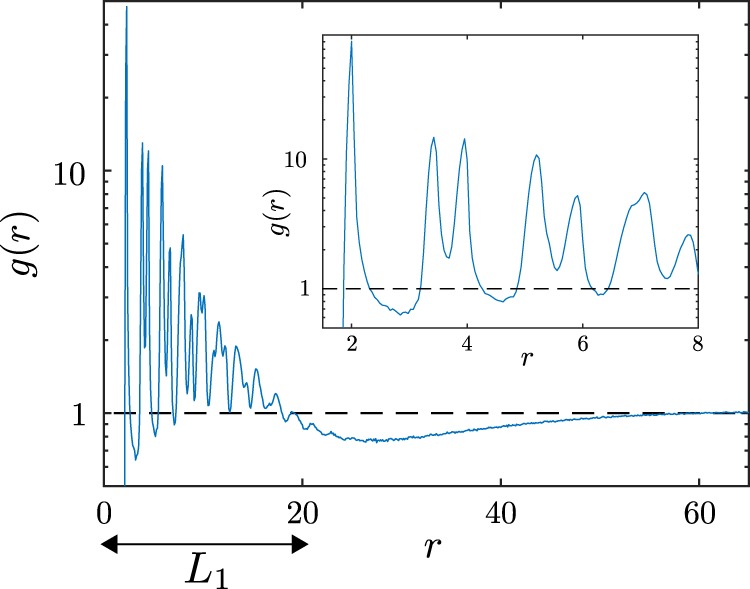
Pair-correlation function $g(r)$ (radial average of $g({\bf{r}})$) of a system of $2N=2000$ particles at time $t=250$. The data are averaged over 100 independent ensembles. The dashed line shows a threshold to extract a characteristic length scale. Parameters as in Fig. 2(d).

Having explored how the patterns emerge and behave at early times, we now want to explore their structure and dynamics also at late times. To do this, we introduce the instantaneous pair-correlation function $g({\bf{r}})$ defined as

9 $$g({\bf{r}})=\frac{1}{{\rho }_{{\rm{id}}}}\left\langle {\sum }_{i\ne 0}\delta ({\bf{r}}-{{\bf{r}}}_{i})\right\rangle ,$$

for an average number density ${\rho }_{{\rm{id}}}=\frac{2N}{{L}_{{\rm{box}}\,}^{2}}$ with box length ${L}_{{\rm{box}}}=250$, total number of particles $2N$ and $\langle \cdot \rangle$ denoting the ensemble average. The radially averaged and time averaged pair-correlation function $g(r)$, where $r=| {\bf{r}}|$, shown in Fig. 7 describes how the density varies as a function of distance from a reference particle at which we averaged over all particles of hunters and prey.

As one can see in the inset of Fig. 7, there is a large peak around $r=2$, which is the typical distance between two particles ($R=1$ in dimensionless units). We can also find peaks around $r=2\sqrt{3}$ and $r=4$ caused by the next two neighbors. This reflects the fact that the static clusters (blue, orange, red) show a hexagonal packing.

### Late-stage dynamics

**Figure 8:**
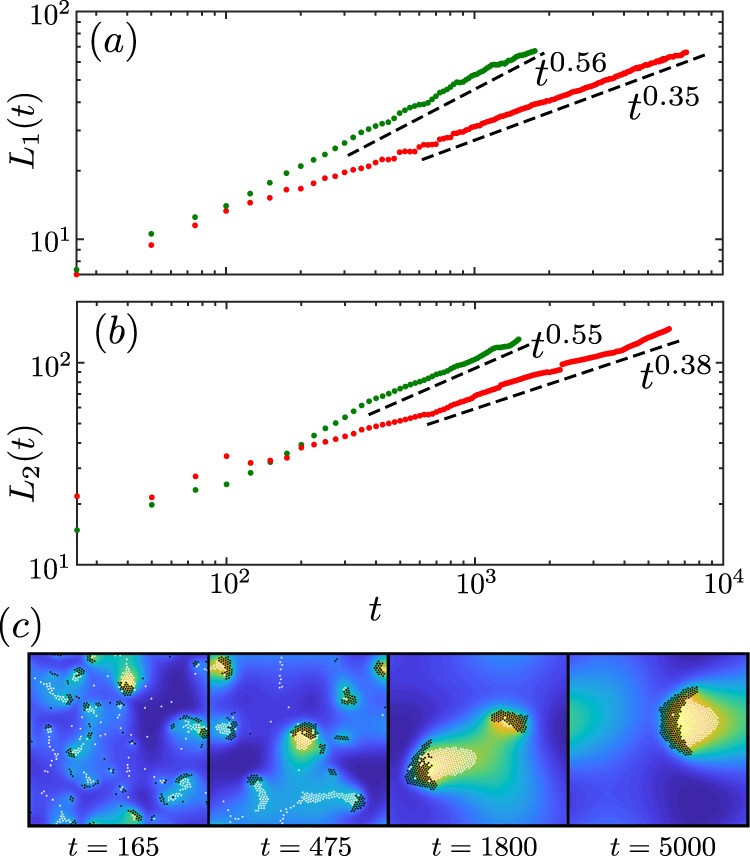
Time-dependent characteristic length scale (**a**) obtained from the pair-correlation function and (**b**) from the structure factor for structures in the red region (red dotted line) and in the green region (green dotted line of Fig. 3(a)). The dashed lines indicate the fitted exponents. Parameters as in Fig. 2(a,d). Panel **(c)** shows a sequence of snapshots from a representative simulation of the hunting swarms which continuously collide, split up and grow to a larger size.

Once the patterns have emerged, they reach a state where their morphology changes only slowly. However, even at late stages the size of the individual structures still increases in time. The swarms move ballistically and frequently collide with each other often leading to their break up. They still grow on average, ultimately leading to a single swarm at late times. This is because after each collision a new swarm forms rapidly, i.e. on timescales before the individual particles which were part of the ‘old’ swarm significantly diffuse away. The newly forming swarm rapidly reaches a size exceeding that of its “ancestors”, because it involves particles from both swarms which were involved in the collision. To quantify this growth, we consider the time evolution of the radial distribution function $g(r,t)$ and define the length scale ${L}_{1}(t)$ of clusters as the smallest value where $g(r,t)\le 1$, for all $r > {L}_{1}$. Thus, the $g(r)$ shown in Fig. 7 corresponds to a length scale of ${L}_{1}\approx 20$ (dimensionless units). At late-times, we find that ${L}_{1}(t)\propto {t}^{\beta }$ follows a power law with an exponent of $\beta \approx 0.35$ (Fig. 8) for the (nonmoving) cluster phases, which is close to the value of $\beta =\frac{1}{3}$ as expected for diffusive growth (in the absence of hydrodynamic interactions)^43–47^. We find a much larger exponent, of $\beta \approx 0.56$ (Fig. 8), for the patterns in the green region, which is close to $\beta =0.5$ as expected for ballistic aggregation. This is a consequence of the fact that the individual structures move ballistically, collide and merge with each other much faster (but also break up).

**Figure 9:**
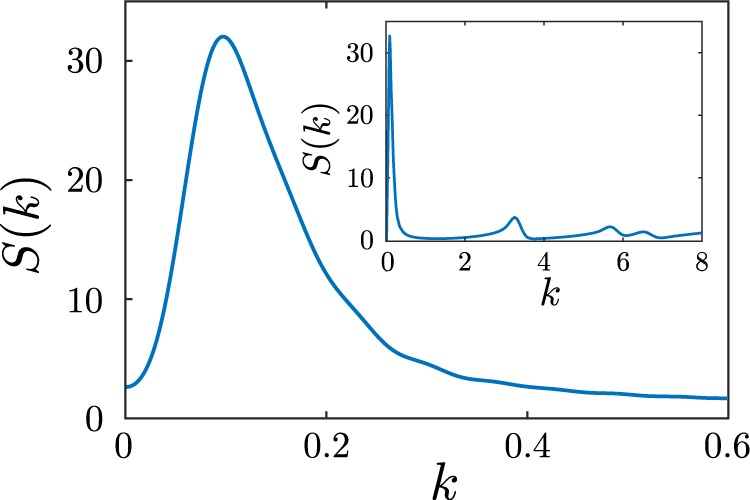
Small wavenumber regime of the structure factor $S(k)$ for a system of $2N=2000$ particles at time $t=250$. Inset shows $S(k)$ for a larger wavenumber regime. The wavenumber $k$ is given in dimensionless units (${k}_{0}=\frac{2\pi }{R}$). Parameters as in Fig. 2(d).

As a second measure for the growth of the clusters, we measure the distance between them. To do this, we consider the structure factor of the system:

10 $$S({\bf{k}})=1+{\rho }_{id}{\int }_{V}{\rm{d}}{\bf{r}}{e}^{-i{\bf{k}}\cdot {\bf{r}}}[g({\bf{r}})-1]$$

and calculate the distance between clusters as the inverse of the first moment of the structure factor^48^, i.e. as:

11 $${L}_{2}(t)=2\pi {\left[\frac{{\int }_{2\pi /L}^{{k}_{cut}}kS(k,t){\rm{d}}k}{{\int }_{2\pi /L}^{{k}_{cut}}S(k,t){\rm{d}}k}\right]}^{-1},$$

where we choose the cutoff wavelength ${k}_{cut}$ as the first local minimum of $S(k)$^48^. Figure 9 shows the structure factor for a cluster in the red region of Fig. 3(a) at time $t=250$ for small values of $k$. The peaks that can be seen in the inset of Fig. 9 correspond to the distance of two possible lattice planes of the hexagonal structure. The peak at $k=3.3$ results from the minimum distance between two particles $\left(\frac{2\pi }{3.3}\approx 2\right)$. One finds a huge peak around $k=0.11$ with which we can estimate a typical length, $l\approx \frac{2\pi }{k}=57.1$; the enormous size of the peak hinges on the fact that each of the contributing clusters contains a large number of particles. The $k$-value where this peak occurs corresponds to the mean cluster distance, which corresponds to the value of $r$ where $g(r)$ approaches 1 from below (see Fig. 7). This distance grows basically with the same power law as the cluster sizes, as shown in Fig. 8, i.e. calculating cluster sizes via ${L}_{1}(t)$ and calculating cluster-distances ${L}_{2}(t)$ basically leads to the same growth law (Fig. 8)^49^. Thus, there is only one independent macroscopic length scale in the system.

## Conclusions

Inspired by the generic presence of multi-species chemotaxis in microbiological communities, e.g. in macrophage-tumor cell systems, we have proposed and explored a physical minimal model to study the collective behaviour beyond the commonly considered one-species limit. We have found that the novel key ingredient of our model - the species selective chemical production - leads to interesting behavior: patterns that comprise a "hunting swarm” phase consisting of a crowd of particles of one species pursuing the other species, and a phase where the two-species self-aggregate in a core-shell structure, which then dissolves abruptly in a dynamic process by ejecting the inner particles.

All these patterns could be observed both on the level of a particle-based description (Eqs. (3), (4)) and in a continuum model (Eqs. (5), (6)), allowing to analytically understand the transition line between cluster phases, which originate from a stationary instability of the uniform phase, and hunting swarms, emerging from an oscillatory instability. As a further characteristic difference between these phases, we find that clusters (and the distance between them) grow diffusively ($L(t)\propto {t}^{0.35}$)^43–47,49^, whereas hunting swarms grow significantly faster ($L(t)\propto {t}^{0.56}$)^50^.

While the key aim of the present work was to explore a minimal framework illustrating how chemical cross-interactions may lead to complex behavior, it should in principle be possible to realize the present model also with (autophoretic) colloidal mixtures, e.g. based on a combination of nonreciprocal repulsive thermo-phoretic and attractive chemo-phoretic interactions, which have been confirmed to be non-reciprocal in recent experiments^29^.

Future work might include more specific biological details and could address the effect of confining boundaries or obstacles^51–54^. Other topics concern additional aligning interactions and their impact on the cluster structure^55–57^ and ternary systems describing species of a longer biological food chain.

## Supplementary information

Supplementary Movie 1.

Supplementary Movie 2.

Supplementary Movie 3.

Supplementary Movie 4.

Supplementary Movie 5.

Supplementary Movie 6.

Supplementary Movie 7.

Supplementary Movie 8.

Supplementary Movie 9.

Supplementary Movie 10.

Supplementary Information.

## Data Availability

All relevant data are available from the authors upon reasonable request.

## References

[1] Čejková, J., Holler, S., Nguyenová, T. Q., Kerrigan, C., Štěpánek, F. & Hanczyc, M. M. In *Advances in Unconventional Computing* (Springer, 2017)

[2] Wadhams GH, Armitage JP (2005). Making sense of it all: bacterial chemotaxis. Nat. Rev. Mol. Cell Biol..

[3] Eidi Z, Mohammad-Rafiee F, Khorrami M, Gholami A (2017). Modelling of Dictyostelium discoideum movement in a linear gradient of chemoattractant. Soft Matter.

[4] Laganenka, L., Colin, R. & Sourjik, V. Chemotaxis towards autoinducer 2 mediates autoaggregation in Escherichia coli *Nat. Commun*. **7**, 12984 EP (2016).article 10.1038/ncomms12984 PMC5056481 27687245

[5] Tindall MJ, Maini PK, Porter SL, Armitage JP (2008). Overview of mathematical approaches used to model bacterial chemotaxis II: bacterial populations. Bull. Math. Biol..

[6] Murray, J. D. Bacterial Patterns and Chemotaxis, In *Mathematical Biology: II: Spatial Models and Biomedical Applications*, edited by Murray, J. D. (Springer New York, New York, NY, 2003)

[7] Hillen T, Painter KJ (2008). A user’s guide to PDE models for chemotaxis. J. Math. Biol..

[8] Painter, K. J. Mathematical models for chemotaxis and their applications in self-organisation phenomena *J. Theor. Biol*. (2018). 10.1016/j.jtbi.2018.06.019 29944856

[9] Painter KJ, Hillen T (2011). Spatio-temporal chaos in a chemotaxis model. Phys. D.

[10] Dolak Y, Schmeiser C (2005). Kinetic models for chemotaxis: Hydrodynamic limits and spatio-temporal mechanisms. J. Math. Biol..

[11] Mukherjee M, Ghosh P (2018). Growth-mediated autochemotactic pattern formation in self-propelling bacteriaC. Phys. Rev. E.

[12] Bergmann F, Rapp L, Zimmermann W (2018). Active phase separation: A universal approach. Phys. Rev. E.

[13] Knútsdóttir H, Palsson E, Edelstein-Keshet L (2014). Mathematical model of macrophage-facilitated breast cancer cells invasion. J. Theor. Biol..

[14] Stark H (2018). Artificial Chemotaxis of Self-Phoretic Active Colloids: Collective Behavior. Acc. Chem. Res..

[15] Robertson B, Huang M-J, Chen J-X, Kapral R (2018). Synthetic Nanomotors: Working Together through Chemistry. Acc. Chem. Res..

[16] Liebchen B, Löwen H (2018). Synthetic Chemotaxis and Collective Behavior in Active Matter. Acc. Chem. Res..

[17] Marchetti MC (2013). Hydrodynamics of soft active matter. Rev. Mod. Phys..

[18] Romanczuk P, Bär M, Ebeling W, Lindner B, Schimansky-Geier L (2012). Active Brownian particles. Eur. Phys. J.-Spec. Top..

[19] Kurzthaler, C. *et al*. Probing the Spatiotemporal Dynamics of Catalytic Janus Particles with Single-Particle Tracking and Differential Dynamic Microscopy *Phys. Rev. Lett*. **121**, 078001 (2018). 10.1103/PhysRevLett.121.078001 30169062

[20] Bechinger C (2016). Active particles in complex and crowded environments. Rev. Mod. Phys..

[21] Aranson IS (2013). Active colloids. Phys.-Usp.

[22] Saha S, Golestanian R, Ramaswamy S (2014). Clusters, asters, and collective oscillations in chemotactic colloids. Phys. Rev. E.

[23] Pohl O, Stark H (2014). Dynamic Clustering and Chemotactic Collapse of Self-Phoretic Active Particles. Phys. Rev. Lett..

[24] Liebchen B, Marenduzzo D, Pagonabarraga I, Cates M (2015). Clustering and Pattern Formation in Chemorepulsive Active Colloids. Phys. Rev. Lett..

[25] Liebchen B, Marenduzzo D, Cates M (2017). Phoretic Interactions Generically Induce Dynamic Clusters and Wave Patterns in Active Colloids. Phys. Rev. Lett..

[26] Huang M-J, Schofield J, Kapral R (2017). Chemotactic and hydrodynamic effects on collective dynamics of self-diffusiophoretic Janus motors. New J. Phys.

[27] Liebchen B, Löwen H (2019). Which interactions dominate in active colloids?. J. Chem. Phys..

[28] Soto R, Golestanian R (2014). Self-Assembly of Catalytically Active Colloidal Molecules: Tailoring Activity Through Surface Chemistry. Phys. Rev. Lett..

[29] Schmidt F, Liebchen B, Löwen H, Volpe G (2019). Light-controlled assembly of active colloidal molecules. J. Chem. Phys..

[30] Niu, R., Palberg, T. & Speck, T. Self-Assembly of Colloidal Molecules due to Self-Generated Flow *Phys. Rev. Lett*. **119**, 028001 (2017). 10.1103/PhysRevLett.119.028001 28753375

[31] Stürmer J, Seyrich M, Stark H (2019). Chemotaxis in a binary mixture of active and passive particles. J. Chem. Phys..

[32] Singh DP, Choudhury U, Fischer P, Mark AG (2017). Non-Equilibrium Assembly of Light-Activated Colloidal Mixtures. Advanced Materials.

[33] Agudo-Canalejo J, Golestanian R (2019). Active Phase Separation in Mixtures of Chemically Interacting Particles. Phys. Rev. Lett..

[34] Wang L, Popescu MN, Stavale F, Ali A, Gemming T, Simmchen J (2018). Cu@TiO$${}_{2}$$ Janus microswimmers with a versatile motion mechanism. Soft Matter.

[35] Ivlev A, Bartnick J, Heinen M, Du C-R, Nosenko V, Löwen H (2015). Statistical mechanics where Newton’s third law is broken. Phys. Rev. X.

[36] Sengupta A, Kruppa T, Löwen H (2011). Chemotactic predator-prey dynamics. Phys. Rev. E.

[37] Boonman, A., Yovel, Y. & Fenton, B. The benefits of insect-swarm hunting in echolocating bats, and its influence on the evolution of bat echolocation signals, bioRxiv, 554055 (2019). 10.1371/journal.pcbi.1006873 PMC6907744 31830029

[38] Jeschke JM, Tollrian R (2007). Prey swarming: which predators become confused and why?. Animal Behaviour.

[39] Zhdankin V, Sprott JC (2010). Simple predator-prey swarming model. Phys. Rev. E.

[40] Angelani L (2012). Collective Predation and Escape Strategies. Phys. Rev. Lett..

[41] Hauke, F., Löwen, H. & Liebchen, B. Clustering-induced velocity-reversals of active colloids mixed with passive particles. *J. Chem. Phys.* **152**, 014903 (2020). 10.1063/1.5128641 31914737

[42] Aranson IS, Kramer L (2002). The world of the complex Ginzburg-Landau equation. Rev. Mod. Phys..

[43] Lifshitz I, Slyozov V (1961). The kinetics of precipitation from supersaturated solid solutions ☆. J. Phys. Chem. Solids.

[44] Bray AJ (2002). Theory of phase-ordering kinetics. Adv. Phys..

[45] Gonnella G, Marenduzzo D, Suma A, Tiribocchi A (2015). Motility-induced phase separation and coarsening in active matter. C. R. Phys..

[46] Laradji M, Kumar PBSunil (2005). Domain growth, budding, and fission in phase-separating self-assembled fluid bilayers. J. Chem. Phys..

[47] Camley BA, Brown FLH (2011). Dynamic scaling in phase separation kinetics for quasi-two-dimensional membranes. J. Chem. Phys..

[48] Stenhammar J, Tiribocchi A, Allen RJ, Marenduzzo D, Cates ME (2013). Continuum Theory of Phase Separation Kinetics for Active Brownian Particles. Phys. Rev. Lett..

[49] Stanich C (2013). Coarsening Dynamics of Domains in Lipid Membranes. Biophys. J..

[50] Cremer P, Löwen H (2014). Scaling of cluster growth for coagulating active particles. Phys. Rev. E.

[51] Morin, A., Desreumaux, N., Caussin, J.-B. & Bartolo, D. Distortion and destruction of colloidal flocks in disordered environments *Nat. Phys*.**13**, 63 EP (2016).

[52] Toner J, Guttenberg N, Tu Y (2018). Hydrodynamic theory of flocking in the presence of quenched disorder. Phys. Rev. E.

[53] Huang M-J, Schofield J, Kapral R (2017). Transport in active systems crowded by obstacles. J. Phys. A: Math. Theor.

[54] Rahmani, P., Peruani, F. & Romanczuk, P. Flocking in complex environments – attention trade-offs in collective information processing, arXiv:1907.11691 [physics.bio-ph] (2019). 10.1371/journal.pcbi.1007697 PMC7173936 32251423

[55] Das SK (2017). Pattern, growth, and aging in aggregation kinetics of a Vicsek-like active matter model. J. Chem. Phys..

[56] Mones E, Czirók A, VicsekAnomalous T (2015). segregation dynamics of self-propelled particles. New J. Phys..

[57] Nilsson S, Volpe G (2017). Metastable clusters and channels formed by active particles with aligning interactions. New J. Phys..

