# Supplementary material for: Swarm Hunting and Cluster Ejections in Chemically Communicating Active Mixtures
Source: Sci Rep. 2020 Mar 27;10:5594. doi: 10.1038/s41598-020-62324-0 (PMC7101431; doi:10.1038/s41598-020-62324-0)
Supplement: Supplementary file 11 — Supplementary Information. [file 41598_2020_62324_MOESM11_ESM.pdf]

Supplemental Material - Swarm Hunting and Cluster Ejections in Chemically Communicating Active Mixtures

I. DIMENSIONLESS EQUATIONS

In order to extract the essential parameters controlling the behaviour if the considered model, we non-dimensionalize it and introduce *characteristic units*.

A. PDEs for chemotaxis

Every physical quantity is written as a product of a non-dimensional quantity times a normalization constant containing the units, e.g.

$$\mathbf{x} = \tilde{\mathbf{x}}\, x_u \;, \quad t = \tilde{t}\, t_u \tag{1}$$

Here, all the quantities with a tilde are dimensionless while those with a subscript " $_u$ " contain the units. We also introduce the dimensionless chemical field, $\tilde{c}^{p,h} = c^{p,h} x_u^d$, where d denotes the spatial dimension ($d = 2$ in our case). Eq. (2) in the main text then reads in dimensionless form as

$$\partial_{\tilde{t}} \frac{\tilde{c}^s}{x_u^d} = \frac{D_c t_u}{x_u^2} \tilde{\Delta} \frac{\tilde{c}^s}{x_u^d} - k_d t_u \frac{\tilde{c}^s}{x_u^d} + \frac{k_0 t_u}{x_u^d} \sum_{i=1}^{N} \delta(\tilde{\mathbf{r}} - \tilde{\mathbf{r}}_i^s) \;, \quad s \in \{p, h\} \;, \tag{2}$$

where we have used that $\delta(\alpha \mathbf{r}) = \frac{1}{\alpha^d}\delta(\mathbf{r})$. Next, we choose $x_0 = R$, where R denotes the (soft) radius of a particle and $t_u = \frac{1}{k_0}$ yielding:

$$\partial_{\tilde{t}} \tilde{c}^s = \tilde{D}_c \tilde{\Delta} \tilde{c}^s - \mu \tilde{c}^s + \sum_{i=1}^{N} \delta(\tilde{\mathbf{r}} - \tilde{\mathbf{r}}_i^s) \;, \tag{3}$$

where the normalised coefficients $\tilde{D}_c$ and μ are then given by: $\tilde{D}_c = \frac{D_c}{k_0 R^2}$ and $\mu = \frac{k_d}{k_0}$.

B. Particle ODEs

The same normalization is applied to Eq. (1) in the main text which leads to:

$$\partial_{\tilde{t}} \tilde{\mathbf{r}}_i^p = \frac{t_u}{\gamma x_u^{d+2}} \left(\alpha_{pp} \tilde{\nabla} \tilde{c}^p(\tilde{\mathbf{r}}_i^p, t) + \alpha_{ph} \tilde{\nabla} \tilde{c}^h(\tilde{\mathbf{r}}_i^p, t) \right) - \tilde{\nabla} \tilde{V} + \sqrt{\frac{2 D t_u}{x_u^2}} \tilde{\boldsymbol{\eta}}_i^p \tag{4}$$

$$\partial_{\tilde{t}} \tilde{\mathbf{r}}_i^h = \frac{t_u}{\gamma x_u^{d+2}} \left(\alpha_{hh} \tilde{\nabla} \tilde{c}^h(\tilde{\mathbf{r}}_i^h, t) + \alpha_{hp} \tilde{\nabla} \tilde{c}^p(\tilde{\mathbf{r}}_i^h, t) \right) - \tilde{\nabla} \tilde{V} + \sqrt{\frac{2 D t_u}{x_u^2}} \tilde{\boldsymbol{\eta}}_i^h, \tag{5}$$

where $\boldsymbol{\eta}_i^s(t)$ represents unit-variance Gaussian white noise with zero mean. With the additional definitions of $\tilde{\alpha}_{kl} = \frac{\alpha_{kl}}{\gamma k_0 R^{d+2}}$ and $\tilde{D} = \frac{D}{k_0 R^2}$ we obtain:

$$\partial_{\tilde{t}} \tilde{\mathbf{r}}_i^p = \tilde{\alpha}_{pp} \tilde{\nabla} \tilde{c}^p(\tilde{\mathbf{r}}_i^p, t) + \tilde{\alpha}_{ph} \tilde{\nabla} \tilde{c}^h(\tilde{\mathbf{r}}_i^p, t) - \tilde{\nabla} \tilde{V} + \sqrt{2\tilde{D}} \tilde{\boldsymbol{\eta}}_i^p \tag{6}$$

$$\partial_{\tilde{t}} \tilde{\mathbf{r}}_i^h = \tilde{\alpha}_{hh} \tilde{\nabla} \tilde{c}^h(\tilde{\mathbf{r}}_i^h, t) + \tilde{\alpha}_{hp} \tilde{\nabla} \tilde{c}^p(\tilde{\mathbf{r}}_i^h, t) - \tilde{\nabla} \tilde{V} + \sqrt{2\tilde{D}} \tilde{\boldsymbol{\eta}}_i^h. \tag{7}$$

We model V using the Weeks-Chandler-Anderson potential, with $\tilde{V} = \frac{1}{2}\sum_{i,j\neq i}\tilde{V}_{ij}$, where the sums run over all particles and where

$$\tilde{V}_{ij} = \begin{cases} 4\tilde{\epsilon}\left[(\frac{\tilde{\sigma}}{\tilde{r}_{ij}})^{12} - (\frac{\tilde{\sigma}}{\tilde{r}_{ij}})^{6}\right] + \tilde{\epsilon} & \text{if } \tilde{r}_{ij} \leq 2^{1/6}\tilde{\sigma} \\ 0 & \text{if } \tilde{r}_{ij} > 2^{1/6}\tilde{\sigma} \end{cases} \tag{8}$$

Here r_{ij} denotes the distance between particle i and j, $\tilde{r}_{ij} = r_{ij}/x_u$, $\tilde{\sigma} = \sigma/x_u$ and $\tilde{\epsilon} = \frac{\epsilon}{\gamma k_0 R^2}$.

In the following, we omit tildes. The specific value of ϵ hardly affects our results, so the following independent and relevant dimensionless parameters remain:

1) effective diffusion constants D_c and D,
2) reduced coupling constants α_{kl},
3) ratio of decay and production of the chemical μ
4) density of species p,h (which is conserved throughout the dynamics)

II. DESCRIPTION OF NUMERICAL METHODS

A. Numerical methods for solving the equations of the particle-based chemotaxis model

We solve our model Eqs. (3,6,7) by discretizing space and time. We perform simulations in two spatial dimensions (2D) with periodic boundary conditions. Interactions between the particles are described using the Weeks-Chandler-Anderson potential. The PDEs (3) for the dynamics of the chemical fields are solved in time with a forward Euler method. This does not impose any restriction on the timestep dt due to the Courant-Friedrich-Lewy (CFL) condition [1] of the heat equation ($D_c\left(\frac{dt}{dx^2} + \frac{dt}{dy^2}\right) < \frac{1}{4}$, where dt and dx, dy denote the temporal and spatial discretisations), since the necessary timestep dt for the particle dynamics is small enough to not violate the above CFL condition. The Laplace operator of the heat term is approximated with central differences. The particle dynamics of the colloids (Eqs. 6,7) is solved in time with the Euler-Maruyama scheme [2] for incorporation the noise term. The interactions of the colloids is effectively treated by applying a cell-list summation [3].

In order to speed up the calculations, we make use of cell lists [3], with a minimal image convention. This is motivated by the fact that the repulsive force caused by the potential V is short ranged and hence we can introduce a cutoff radius $r_c = 2^{1/6}\sigma$. More explicitly, we consider only interactions within a cutoff radius r_c such that the pair interaction $V_{ij} = 0$ for $r_{ij} > r_c$.

Different simulations are performed by varying the diffusion constants D and D_c, the coupling constants α_{kl} and the ratio of decay and production of the chemical μ.

Typical parameters used in the simulation of the dimensionless Eqs. (3,6,7):

# of particles N	$2 * 10^3$
WCA-potential amplitude ϵ	1
coupling constants	$-10 < \alpha_{kl} < 10$
diffusion constant D_c	1
diffusion constant D	$0.001 - 1$
decay/production of c	$0.001 < \mu < 1$
time step	$\Delta t = 0.001 t_0$
simulation time	$\sim 10^4 t_0$
grid for chemical	250 x 250 grid cells

B. Numerical methods for solving the equations of the continuum chemotaxis model

In addition to the particle-based simulations, we have also numerically solved the system of the four coupled dimensionless PDEs of the continuum model, Eqs. (5,6) of the main text as before in two spatial dimensions with periodic boundary conditions. We use a forward Euler method to propagate the densities ρ^s and the chemical fields c^s in time. The Laplace operator is approximated with central differences.

1. Repulsion between cells

We must be careful when implementing the chemotactic continuum model, which unlike our particle based equations, neglect the steric repulsions among the particles, preventing sharp density peaks, destabilizing the simulation. In view of this effect, we add an additional nonlinear term to the continuum model to effectively describe local repulsions among the particles [4]:

$$\partial_t \rho^s = D\Delta\rho^s - \sum_{s'\in\{p,h\}} \alpha_{ss'} \nabla \cdot (\rho^s \nabla c^{s'}) + D^s_{\text{rep}} \nabla \cdot (\rho^s \nabla(\rho^p + \rho^h)) \,. \tag{9}$$

Here, D^s_{rep} with $s \in \{p, h\}$ are dimensionless repulsion coefficients and we assume that the strength of the repulsion is the same between the two different species, $D^p_{\text{rep}} = D^h_{\text{rep}}$.

2. Regularization of the density

As a second adjustment of the continuum model, avoiding a blow up [5] we use:

$$\partial_t \rho^s = D\Delta\rho^s - \sum_{s'\in\{p,h\}} \alpha_{ss'} \nabla \cdot \left(\frac{\rho^s}{1+\kappa\rho^s} \nabla c^{s'} \right) + D^s_{\text{rep}} \nabla \cdot (\rho^s \nabla(\rho^p + \rho^h)) \,, \tag{10}$$

where κ is a small regularization parameter and $\kappa \to 0$ leads to the original system. In [5] it was shown that the solutions of equations of the kind of Eq. (5) of the main text typically have a spiky structure and Eq. (10) can be used to model the aggregation phenomena.

III. LINEAR STABILITY ANALYSIS

To understand the origin of the observed pattern formation, we perform a linear stability analysis of Eqs. (5,6) of the main text around the homogeneous solution $(\rho^s, c^s) = (\rho^s_0, \rho^s_0/\mu) =: (\rho^s_0, c^s_0)$, $s \in \{p, h\}$. The PDEs of the density in the continuous model for two different sorts of particles $\rho^s = \rho^s(\mathbf{r}, t)$, $s \in \{p, h\}$ read (in dimensionless units)

$$\partial_t \rho^p = D\Delta\rho^p - \alpha_{pp} \nabla \cdot (\rho^p \nabla c^p) - \alpha_{ph} \nabla \cdot (\rho^p \nabla c^h) \tag{11}$$

$$\partial_t \rho^h = D\Delta\rho^h - \alpha_{hh} \nabla \cdot (\rho^h \nabla c^h) - \alpha_{hp} \nabla \cdot (\rho^h \nabla c^p) \,, \tag{12}$$

where the corresponding PDEs for the phoretic field $c^s = c^s(\mathbf{r}, t)$, $s \in \{p, h\}$ are given by:

$$\partial_t c^s = D_c \Delta c^s + \rho^s - \mu c^s \,. \tag{13}$$

For the linear stability analysis, we consider small perturbations from the homogeneous steady state of the particle density $\rho^s = \rho^s_0 + \delta\rho^s$ and the phoretic field $c^s = c^s_0 + \delta c^s$.

The resulting linearised system of four equations describes the time-evolution of the deviations of the particle density and the chemical field from the homogeneous state $\rho^s = \rho^s_0$ and $c^s = \rho^s_0/\mu$.

$$\partial_t \delta\rho^p = D\Delta\delta\rho^p - \alpha_{pp}\rho_0^p\Delta\delta c^p - \alpha_{ph}\rho_0^p\Delta\delta c^h \tag{14}$$
$$\partial_t \delta\rho^h = D\Delta\delta\rho^h - \alpha_{hh}\rho_0^h\Delta\delta c^h - \alpha_{hp}\rho_0^h\Delta\delta c^p \ , \tag{15}$$

$$\partial_t \delta c^s = D_c\Delta\delta c^s + \delta\rho^s - \mu\delta c^s \ . \tag{16}$$

Since the linearised equations Eqs. (14-16) do not depend explicitly on time, we perform a Fourier transformation in space and make a separation Ansatz $\hat{\rho}^s(\mathbf{q},t) = e^{\lambda t}\hat{\rho^s}(\mathbf{q})$, $\hat{c}^s(\mathbf{q},t) = e^{\lambda t}\hat{c^s}(\mathbf{q})$, leading immediately to the following eigenvalue problem (we set $\alpha_{hh} = 0$ as in the main text):

$$\lambda \begin{pmatrix} \delta\hat{\rho}^p \\ \delta\hat{c}^p \\ \delta\hat{\rho}^h \\ \delta\hat{c}^h \end{pmatrix} = \begin{pmatrix} -D\mathbf{q}^2 & \alpha_{pp}\rho_0^p\mathbf{q}^2 & 0 & \alpha_{ph}\rho_0^p\mathbf{q}^2 \\ 1 & -D_c\mathbf{q}^2 - \mu & 0 & 0 \\ 0 & \alpha_{hp}\rho_0^h\mathbf{q}^2 & -D\mathbf{q}^2 & 0 \\ 0 & 0 & 1 & -D_c\mathbf{q}^2 - \mu \end{pmatrix} \begin{pmatrix} \delta\hat{\rho}^p \\ \delta\hat{c}^p \\ \delta\hat{\rho}^h \\ \delta\hat{c}^h \end{pmatrix} \ , \tag{17}$$

where the stability of the system is determined by the eigenvalues λ of the matrix, as follows:

(a) The steady state is stable, if the eigenvalues of the matrix all have real parts strictly less than zero.

(b) The steady state is unstable, if at least one of the eigenvalues of the matrix has a positive real part.

(c) Otherwise in the marginal case higher order terms determine the stability of the problem.

The four eigenvalues λ of the linearised system are explicitly given by ($\alpha_{hh} = 0$, $\rho_0^p = \rho_0^h = \rho_0$):

$$\lambda = -\frac{q^2}{2}(D_c + D + \frac{\mu}{q^2}) \pm \frac{1}{2}\sqrt{[q^2(D_c + D + \frac{\mu}{q^2})]^2 - 4(D_c + \frac{\mu}{q^2})Dq^4 + 2\alpha_{pp}\rho_0 q^2 \pm 2q^2\rho_0\sqrt{4\alpha_{ph}\alpha_{hp} + \alpha_{pp}^2}} \tag{18}$$

For the parameter range under consideration, eigenvalues with non-vanishing imaginary part exist ($\mathrm{Im}(\lambda) \neq 0$), if the condition $-4\alpha_{ph}\alpha_{hp} > \alpha_{pp}^2$ is fulfilled. In order to develop an instability for long wavelengths ($|q| \longrightarrow 0$), a Taylor series extension of (18) and an examination where $\mathrm{Re}(\lambda) > 0$ provides us with the criterion

$$2D\mu < \alpha_{pp}\rho_0. \tag{19}$$

If the eigenvalues are real (here, $\mathrm{Im}(\lambda) = 0 \iff -4\alpha_{ph}\alpha_{hp} < \alpha_{pp}^2$), then we obtain the following criterion for instability from calculating $\mathrm{Re}(\lambda) > 0$:

$$2D\mu < \rho_0\left(\alpha_{pp} + \sqrt{4\alpha_{ph}\alpha_{hp} + \alpha_{pp}^2}\right) \tag{20}$$

Altogether, an instability is given, when the following criterion is fulfilled:

$$2D\mu < \rho_0 \mathrm{Re}\left[\alpha_{pp} + \sqrt{4\alpha_{ph}\alpha_{hp} + \alpha_{pp}^2}\right] \tag{21}$$

and this instability is also oscillatory if

$$-4\alpha_{ph}\alpha_{hp} > \alpha_{pp}^2 \ . \tag{22}$$

If the local repulsions among the particles is taken into account and the linear stability analysis is performed with Eq. (9) instead of Eq.(5) of the main text, the uniform phase is unstable, when the following criterion is fulfilled:

$$2\mu(D + \rho_0 D_{rep}) < \rho_0 \mathrm{Re}\left[\alpha_{pp} + \sqrt{4\alpha_{ph}\alpha_{hp} + \alpha_{pp}^2 - 4\mu D_{rep}(\alpha_{ph} + \alpha_{hp} - \mu D_{rep})}\right] \tag{23}$$

which is then also oscillatory if

$$-4\alpha_{ph}\alpha_{hp} > \alpha_{pp}^2 - 4\mu D_{rep}(\alpha_{ph} + \alpha_{hp} - \mu D_{rep}) \ . \tag{24}$$

Since the term μD_{rep} is very small for the considered parameters in Fig. 2 (of the main text), the instability criterion is barely influenced by this additional D_{rep}-term approximately leading to Eqs. (21,22) again.

Note that the instability criterion, Eq. (21), does not depend on the chemical diffusion constant D_c. Therefore, a faster alternative to obtain Eq. (21), would be to assume that the dynamics of the chemical is fully enslaved by the motion of the colloids and relaxes quasi-instantaneously to its steady state, i.e. to assume $\partial_t c^s = 0$. Note however, that only the instability criterion itself is independently of D_c and not the instability band. In addition, once the instability has emerged, the fact that $\partial_t c^s \neq 0$ is of crucial importance for the phenomenology of the resulting patterns, which can be seen best for the clusters ejecting their inner particles, which hinge on chemical delay (or memory) effects.

We have numerically tested the instability criterion in the parameter regime where it is oscillatory (i.e. non-vanishing imaginary part) to see if it is shifted due to perturbation convection, see [6]. Snapshots of the additional performed simulations are shown below in Fig. 1. The snapshots are arranged in a row with increasing decay-rate of the chemical μ. Above a certain value, this rate is too large, such that the concentration of the chemical substances is insufficient and the homogeneous phase is stable. The dashed line indicates for which μ the instability criterion (Eq. (7)) is fulfilled. As can be seen, this transition line fits well to the simulations and we did not find any shift, suggesting that the advective and absolute instability are very close to each other in the present case.

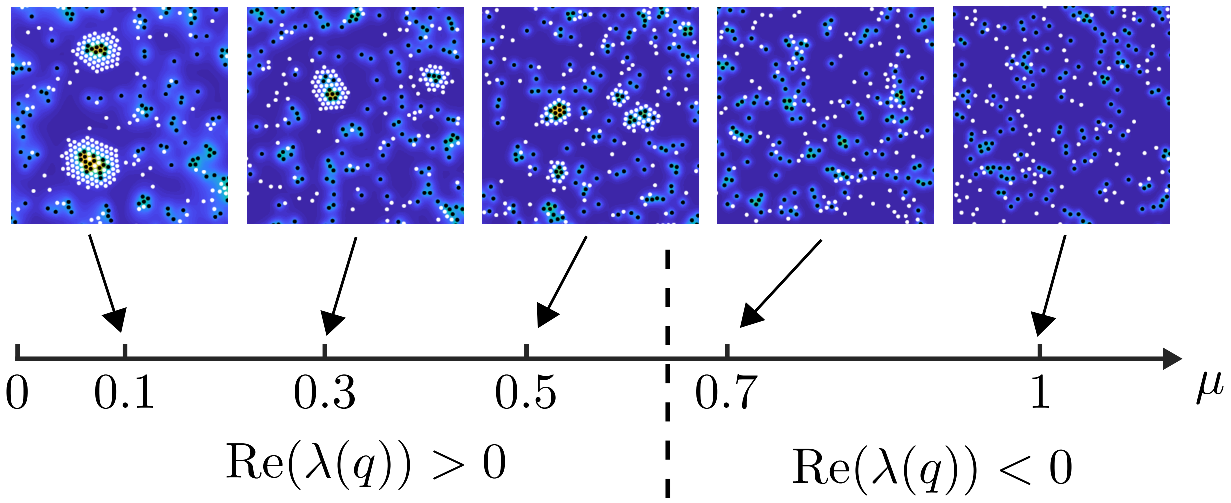

FIG. 1: Snapshots of simulations with different decay-rate μ. The dashed line indicates for which μ the instability criterion is fulfilled. Parameters used in these simulations: $\alpha_{pp} = 1, \alpha_{hh} = 0, \alpha_{ph} = -10, \alpha_{hp} = 10, D = 0.01, N = 2000, L = 250$

IV. MEAN PARTICLE VELOCITY

In order to distinguish between the different phases, in particular between the clusters and the hunting swarm phase, we calculate the mean particle velocity, which is defined as

$$v(t) = \left\langle \frac{1}{2N} \sum_{\substack{i=1,N \\ s=p,h}} \left| \frac{\mathbf{r}_i^s(t+dt) - \mathbf{r}_i^s(t)}{dt} \right| \right\rangle \tag{25}$$

where we average over all particles of species $s \in \{p, h\}$ and $\langle \cdot \rangle$ denotes the ensemble average.

V. DIFFUSION EQUATION IN 3D

To verify that our results do not change qualitatively when solving the diffusion equation in 3D, we have performed additional simulations for verification. Figure 2 shows an exemplaric simulation snapshot of the particle based model in three dimensions for a cluster in the blue domain ($\alpha_{hp} = 0.01, \alpha_{ph} = -10$).

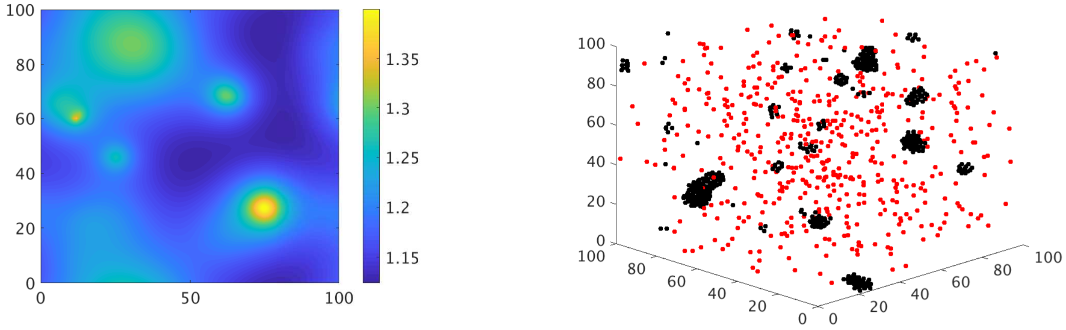

FIG. 2: Simulation snapshot of the particle based model in 3D. Left panel shows the diffusion of c^p by looking at the cross section of the chemical field in a plane perpendicular to the z-axis. Right panel shows the prey and the hunters, colored in black and red, respectively. Parameters as in Fig. 2(b) of the main text.

VI. DESCRIPTION OF THE MOVIES

The movies 1-8 show the time-evolution of the different patterns, hunting swarms, mixed clusters and core-shell clusters, for the particle-based and the continuum model for the same parameters as used in Fig. 2 of the main text (panels (a-h) corresponding to the files mov1-mov8). Movie 9 shows a dynamical inside out reversal for an appropriate initial condition, starting with a dense and comparatively large cluster of prey particles, which is then invaded by the hunter particles. Parameters used for movie 9: $\alpha_{pp} = 1$, $\alpha_{hh} = 0$, $\alpha_{hp} = 10$, $\alpha_{ph} = -0.01$, $\mu = 0.001$, $D_c = 0.1$, $D = 0.003$, $\epsilon = 1$, $2N = 300$ particles and $L_{\text{box}} = 80$. Movie 10 shows clusters ejecting their inner particles that occur starting from a uniform initial state. Parameters used for movie 10: $\alpha_{pp} = 100$, $\alpha_{hh} = 0$, $\alpha_{hp} = 10$, $\alpha_{ph} = -1000$, $\mu^p = 0.1$, $\mu^h = 0.01$, $D_c^p = 0.5$, $D_c^h = 10$, $D = 0.001$, $\epsilon = 10$, $L_{\text{box}} = 200$ with 500 prey-particles and 2000 hunter-particles.

REFERENCES

[1] R. Courant, K. Friedrichs, and H. Lewy, Über die partiellen Differenzengleichungen der mathematischen Physik, Math. Ann. **100**, 32 (1928).

[2] D. J. Higham., An Algorithmic Introduction to Numerical Simulation of Stochastic Differential Equations, SIAM Rev. **43**, 525 (2001).

[3] M. P. Allen and D. J. Tildesley, *Computer Simulation of Liquids* (Clarendon Press, New York, NY, USA, 1989).

[4] H. Knútsdóttir, E. Palsson, and L. Edelstein-Keshet, Mathematical model of macrophage-facilitated breast cancer cells invasion, J. Theor. Biol. **357**, 184199 (2014).

[5] A. Kurganov and M. Lukacova-Medvidova, Numerical study of two-species chemotaxis models, Discrete and Continuous Dynamical Systems. Series B **1** (2014).

[6] I. S. Aranson and L. Kramer, The world of the complex Ginzburg-Landau equation, Rev. Mod. Phys. **74**, 99 (2002).
